# Relative Agreement and Feasibility of Low-Cost Passive Stereophotogrammetry for Nasal Three-Dimensional Surface Imaging: A Comparison of COLMAP and Agisoft Metashape with a Handheld Structured-Light Scanner

**DOI:** 10.3390/bioengineering13080899

**Published:** 2026-08-09

**Authors:** Xinzhi Li, Shiyayun Yan, Ziqian Zhu, Cang Zhang, Yuhang Wu, Fanming Meng, Libing Yun

**Affiliations:** 1 West China School of Basic Medical Sciences and Forensic Medicine, Sichuan University, Chengdu 610041, China; 2 School of Basic Medical Sciences, Xinjiang Medical University, Urumqi 830017, China

**Keywords:** three-dimensional surface imaging, rhinoplasty, passive stereophotogrammetry, method comparison

## Abstract

Portable handheld three-dimensional (3D) scanners have improved facial surface measurement workflows in plastic and reconstructive surgery and have potential applications in preoperative planning, postoperative assessment, and customized implant design. However, the relative agreement of low-cost passive stereophotogrammetry for nasal surface reconstruction remains insufficiently characterized when compared with handheld structured-light scanning. This study evaluated the feasibility and relative surface-deviation performance of two passive stereophotogrammetry-based systems for nasal 3D surface reconstruction in healthy participants. Twenty-five healthy participants were enrolled, and nasal 3D models were reconstructed using Artec Eva, COLMAP, and Agisoft Metashape. COLMAP- and Agisoft Metashape-derived models were aligned to the corresponding Artec Eva models in MeshLab. Geometric deviations were quantified using root mean square (RMS) surface deviation, mean surface distance (MSD), and Hausdorff distance (HD), and regional discrepancies were visualized using surface-distance heat maps. Both passive pipelines produced deviations generally within previously reported ranges for facial or nasal 3D imaging, with median RMS and MSD values below 0.5 mm. Hausdorff distance showed larger local deviations, particularly in the alar and perinasal regions, and was significantly lower for Agisoft Metashape than for COLMAP. These findings suggest that low-cost passive stereophotogrammetry may support standardized nasal surface measurement under controlled acquisition conditions.

## 1. Introduction

Recent advances in three-dimensional (3D) photography have made it an intuitive, efficient tool for facial measurement and documentation in plastic and reconstructive surgery. Three-dimensional surface imaging (3DSI) is now used preoperatively and postoperatively and has been shown to provide objective craniofacial data, particularly for the head and neck [1,2]. It also improves communication between surgeons and patients. As Persing et al. [3] noted, the shift from two-dimensional (2D) to 3D facial imaging addresses key limitations of 2D assessment and provides more objective information for facial evaluation.

Previous clinical studies have reported that patient-specific facial data derived from 3D imaging may support facial plastic surgery. Previous studies have suggested that 3D imaging may support several stages of facial plastic surgery, including preoperative planning, intraoperative guidance, and postoperative outcome assessment [3,4,5]. In cosmetic practice, preoperative and postoperative evaluations are among its most common uses. The adoption of 3D imaging also improves communication between surgeons and patients and is associated with higher patient satisfaction [6,7,8]. 3D data can be used to generate patient-specific predictive nasal models at a 1:1 scale. These models provide a visual and quantitative reference for planning and allow surgeons to assess outcomes objectively during intraoperative decision-making [7,9,10]. Beyond one-to-one models, 3D data can be used to fabricate simple surgical guides that conform to the nasal surface. These guides provide real-time feedback to verify whether intraoperative nasal morphology matches the preoperative simulation and intended outcome [11,12,13]. 3D imaging further enables customized nasal implant fabrication. It captures anatomy more comprehensively than conventional 2D photography. Surgeons can 3D-print precision silicone implants, reducing operator-dependent variability compared with manual sculpting [14].

Despite its potential in rhinoplasty, broad adoption of 3D imaging has been constrained by the high cost and limited portability of nonportable medical imaging systems. Recent advances have introduced compact, cost-effective handheld 3D scanners to plastic surgery. Previous comparative studies have reported that devices such as the Artec Eva and Artec Space Spider show measurement performance comparable to that of nonportable three-dimensional surface imaging systems [15,16]. These developments support more portable and accessible 3D data acquisition in clinical and research settings.

### Related Work

To broaden access to three-dimensional surface imaging, passive stereophotogrammetry has been increasingly considered a low-cost alternative to dedicated 3D scanning hardware. This approach reconstructs 3D models from overlapping two-dimensional photographs using computational algorithms and requires only a standard camera, controlled acquisition conditions, and a computer [17]. Compared with handheld structured-light scanners, passive stereophotogrammetry requires less specialized hardware and may therefore improve the accessibility of 3D surface imaging in controlled research or preclinical settings. However, its performance for nasal surface reconstruction remains insufficiently characterized, particularly when compared with established handheld structured-light scanning workflows.

COLMAP (Version 3.12.0) and Agisoft Metashape (Version 2.2.0) were selected to represent two practically accessible passive stereophotogrammetry workflows with different implementation characteristics. COLMAP was included as a free, open-source multi-view 3D reconstruction pipeline based on Structure-from-Motion and Multi-View Stereo algorithms [18,19]. It provides both graphical and command-line interfaces and can reconstruct 3D geometry from ordered or unordered multi-view image datasets. Agisoft Metashape was selected as a representative commercial photogrammetry workflow. It is a standalone photogrammetry software package that performs photogrammetric processing of digital images and generates 3D spatial data [20]. Its workflow integrates image alignment, point-cloud generation, mesh reconstruction, and texture mapping, which may reduce the amount of manual post-processing required compared with more modular open-source workflows.

Both COLMAP and Agisoft Metashape can be implemented on a Windows-based workstation and were suitable for processing the standardized 39-image datasets used in this study. Their comparison allowed us to evaluate two practical passive reconstruction routes: a free open-source workflow and a commercial integrated workflow with greater automation.

Artec Eva (Artec Europe S.à r.l., Senningerberg, Luxembourg) was used as the reference comparator because it is a previously evaluated structured-light scanner suitable for rapid acquisition of medium-sized facial surfaces and has been used in previous facial and nasal three-dimensional imaging studies [15,16]. Although Artec Space Spider (Artec Europe S.à r.l., Senningerberg, Luxembourg) provides higher nominal accuracy and resolution, it is more suitable for small objects or local high-detail scanning, whereas Artec Eva better matched the present whole-face and nasal acquisition protocol [21,22]. Therefore, the present study used Artec Eva not as an absolute anatomical ground truth, but as an established structured-light scanning comparator to evaluate the relative agreement of low-cost passive stereophotogrammetry workflows.

Accordingly, this study was designed as a relative agreement and feasibility assessment rather than an absolute validation of anatomical accuracy. We compared nasal surface models reconstructed using COLMAP and Agisoft Metashape with corresponding Artec Eva-derived models to evaluate whether low-cost passive stereophotogrammetry can provide nasal 3D surface data in healthy participants under standardized acquisition conditions.

## 2. Materials and Methods

### 2.1. Ethical Statement and Participant Recruitment

All study procedures were conducted according to the principles expressed in the Declaration of Helsinki. Written informed consent was obtained from all participants. This study was approved by the Medical Ethics Committee of Chengdu Second People’s Hospital on 22 January 2026 ([KY]PJ2026025).

Twenty-five healthy volunteers (13 males and 12 females; mean age 21 ± 2 years) were recruited from Chengdu Second People’s Hospital. Exclusion criteria were prior facial surgery, craniofacial malformations, and a diagnosis or history of epilepsy. No participants were patients undergoing rhinoplasty, nasal reconstruction, or treatment for nasal deformity.

### 2.2. Workflow of the Study

**Figure 1:**
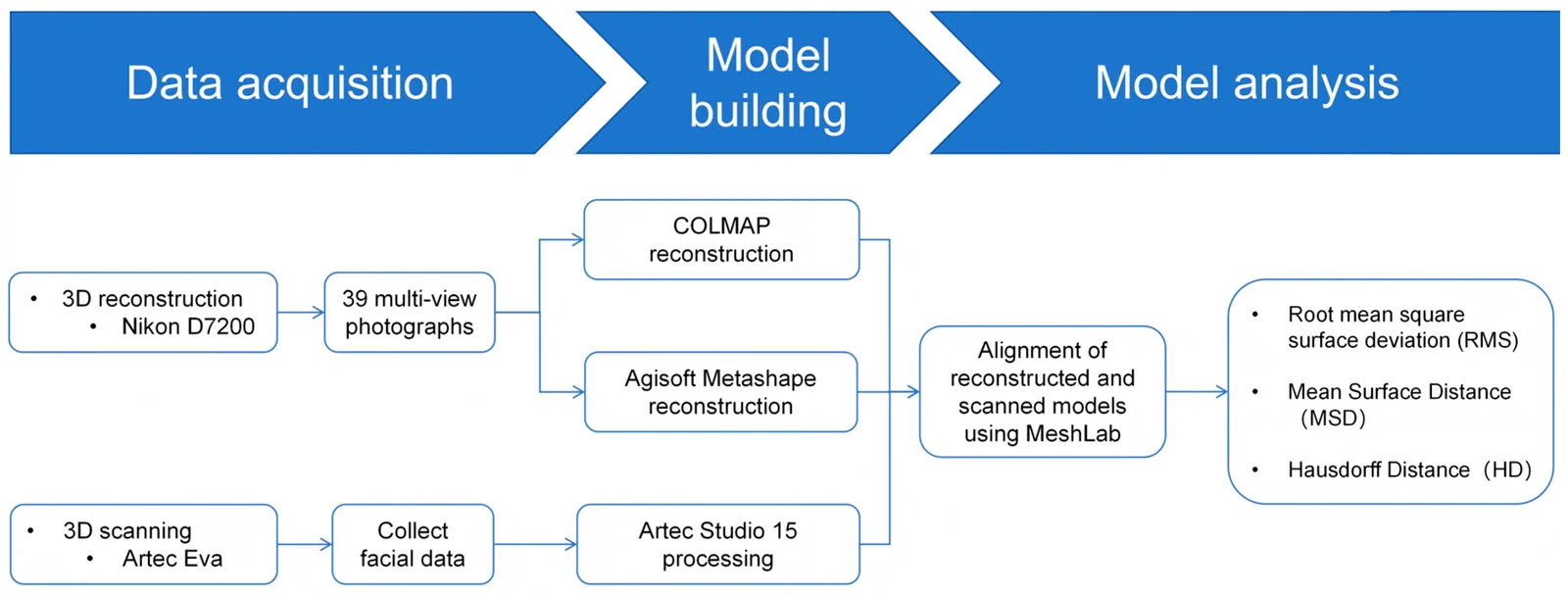
Workflow for comparing the three-dimensional nasal imaging outcomes using three 3D photography systems: Data Acquisition → Model Building → Model Analysis.

The study workflow comprised three phases: data acquisition, model construction, and model analysis (Figure 1). During acquisition, the imaging devices were positioned on a fixed rig directly in front of each subject. To standardize conditions, two fill lights and a black backdrop provided uniform illumination and clear separation from the background. The setup followed the method described by Swamy et al. [23]. To ensure the quality of the final 3D reconstruction, we employed a motorized turntable with a diameter of 1.2 m to maintain consistent distance between the participant and the imaging equipment across all 2D photographs. For 3D reconstruction, 2D images were captured with a Nikon D7200 (Nikon Corporation, Tokyo, Japan) paired with a Sigma 17–50 mm f/2.8 EX DC OS HSM lens (Sigma Corporation, Kawasaki, Japan). Exposure settings were ISO 320, f/8, and 1/30 s. Under these standardized lighting conditions, the configuration provided sufficient detail for facial features. In parallel, an Artec Eva 3D scanner (Artec Europe S.à r.l., Senningerberg, Luxembourg) acquired direct 3D data. The device uses structured-light projection and has been previously evaluated in facial three-dimensional imaging studies [15,24,25].

Prior to imaging, participants removed facial and auricular jewelry and secured hair away from their face to fully expose the region of interest. During acquisition, they maintained natural occlusion, kept a neutral expression with relaxed facial muscles, gazed forward, and sat upright on the same chair mounted on the motorized turntable.

For passive, vision-based 3D reconstruction, we followed the imaging protocol described by Sobral et al. [17]. Building on that protocol, we captured 39 photographs with vertical coverage divided into three segments (upper, middle, and lower), with 13 images per segment. The vertical interval between adjacent segments was approximately 35°. Within each segment, the horizontal sweep spanned 120° (left to right) with approximately 10° between consecutive images. The seventh image in each segment corresponded to the frontal view. The complete 39-image acquisition sequence and photographic coverage for one representative participant are provided in Supplementary Figure S1. For Artec Eva scanning, acquisition covered a horizontal range of approximately 120°, similar to the photographic protocol. This device required imaging in a single horizontal plane to reconstruct a complete 3D model.

**Figure 2:**
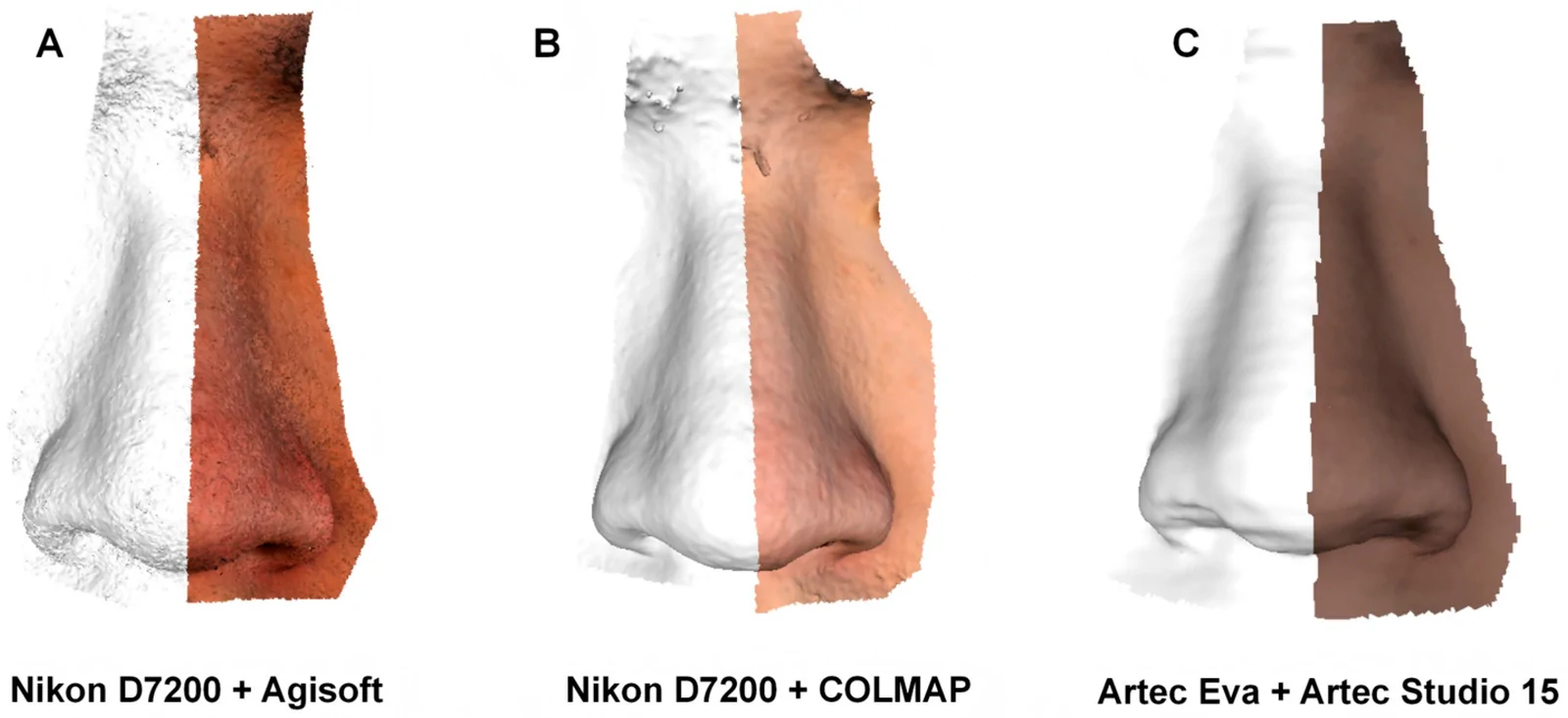
Exemplary nasal 3D models in frontal view. The left panel presents untextured mesh images, and the right panel shows textured mesh images. (**A**) Nasal 3D model reconstructed using Nikon D7200 + Agisoft Metashape. (**B**) Nasal 3D model reconstructed using Nikon D7200 + COLMAP. (**C**) Nasal 3D model reconstructed using Artec Eva + Artec Studio 15.

Two passive stereophotogrammetry-based reconstruction methods were applied to the acquired 2D images to generate 3D surface models. Processing used COLMAP (Version 3.12.0), Agisoft Metashape (Version 2.2.0), and MeshLab (Version 2023.12). For handheld scanning, Artec Studio 15 was used for model construction and refinement. All data processing was conducted on the same Windows 11 workstation equipped with an Intel i5-13400 CPU and an NVIDIA RTX 5060 Ti GPU. All models were exported in the .obj format (Figure 2), a widely supported standard for 3D geometry representation.

For workflow-time assessment, total workflow time, including data acquisition and generation of the final textured 3D model, was recorded for one representative dataset. As the remaining datasets were not systematically timed, the reported durations were treated as approximate descriptive values.

For model analysis, all system-generated models were first scaled to the same size (using the Artec Eva dimensions as the reference). The Iterative Closest Point (ICP) algorithm in MeshLab was then used to register the 3D facial models from COLMAP and Agisoft Metashape to the corresponding Artec Eva model. The ICP algorithm iteratively optimizes rotational and translational transformations to minimize spatial discrepancies between corresponding point clouds [26]. For initial coarse alignment, anatomical landmarks from the Three-Dimensional Cephalometry protocol described by Swennen et al. were manually annotated to achieve gross registration [27]. The software then automatically identified corresponding points and refined the global alignment through ICP iterations until residual errors met the convergence threshold. Following alignment, built-in MeshLab algorithms calculated three surface-deviation metrics between the passive method models and the corresponding structured-light scanner models: Root Mean Square (RMS) surface deviation, Mean Surface Distance (MSD), and Hausdorff Distance (HD). All measurements and analyses were performed independently by two investigators, who were blinded to each other’s results.

The detailed reconstruction, mesh processing, registration, and surface distance calculation procedures for COLMAP, Agisoft Metashape, Artec Studio, and MeshLab are provided in Supplementary File S1.

### 2.3. Data Analysis

Data analysis was conducted using IBM SPSS Statistics 27 for Windows. Interrater reliability of RMS, MSD, and HD measurements obtained by the two independent investigators was evaluated using intraclass correlation coefficients (ICCs). ICCs were calculated using a two-way mixed-effects model with absolute agreement, and average-measure ICCs were reported with 95% confidence intervals because the averaged values from the two investigators were used for subsequent analyses. Box plots were generated to visualize the distributions of RMS, MSD, and HD for COLMAP- and Agisoft Metashape-derived models relative to the Artec Eva comparator. Paired differences between COLMAP and Agisoft Metashape were calculated for each metric, and their normality was assessed using the Shapiro–Wilk test. Because the paired differences did not consistently satisfy the normality assumption, Wilcoxon signed-rank tests were used to compare RMS, MSD, and HD values between COLMAP and Agisoft Metashape. All statistical tests used a two-tailed significance level of 5% (α = 0.05).

To account for the three paired comparisons of HD, RMS, and MSD, *p* values were adjusted using the Holm procedure. Both unadjusted and Holm-adjusted *p* values were reported, and statistical significance was determined using the adjusted *p* values at a two-sided α level of 0.05.

Bland–Altman analyses were performed for RMS, MSD, and HD by plotting the difference between Agisoft Metashape and COLMAP against their mean. Mean bias and 95% limits of agreement were calculated, and the plots are provided in Supplementary Figure S2. Because the paired differences for MSD and HD deviated from normality, the corresponding limits of agreement were interpreted descriptively and with caution.

## 3. Results

### 3.1. General Parameters

All 25 healthy volunteers completed the imaging protocol, and all acquired datasets were included in the final analysis. For each participant, approximately 9 GB of data were collected, including project files and exports from Artec Eva, COLMAP, and Agisoft Metashape. All models were exported in .obj format, yielding 75 3D models for analysis. No reconstruction failures or repeated image acquisitions occurred for any of the three systems.

### 3.2. Workflow Time

**Figure 3:**
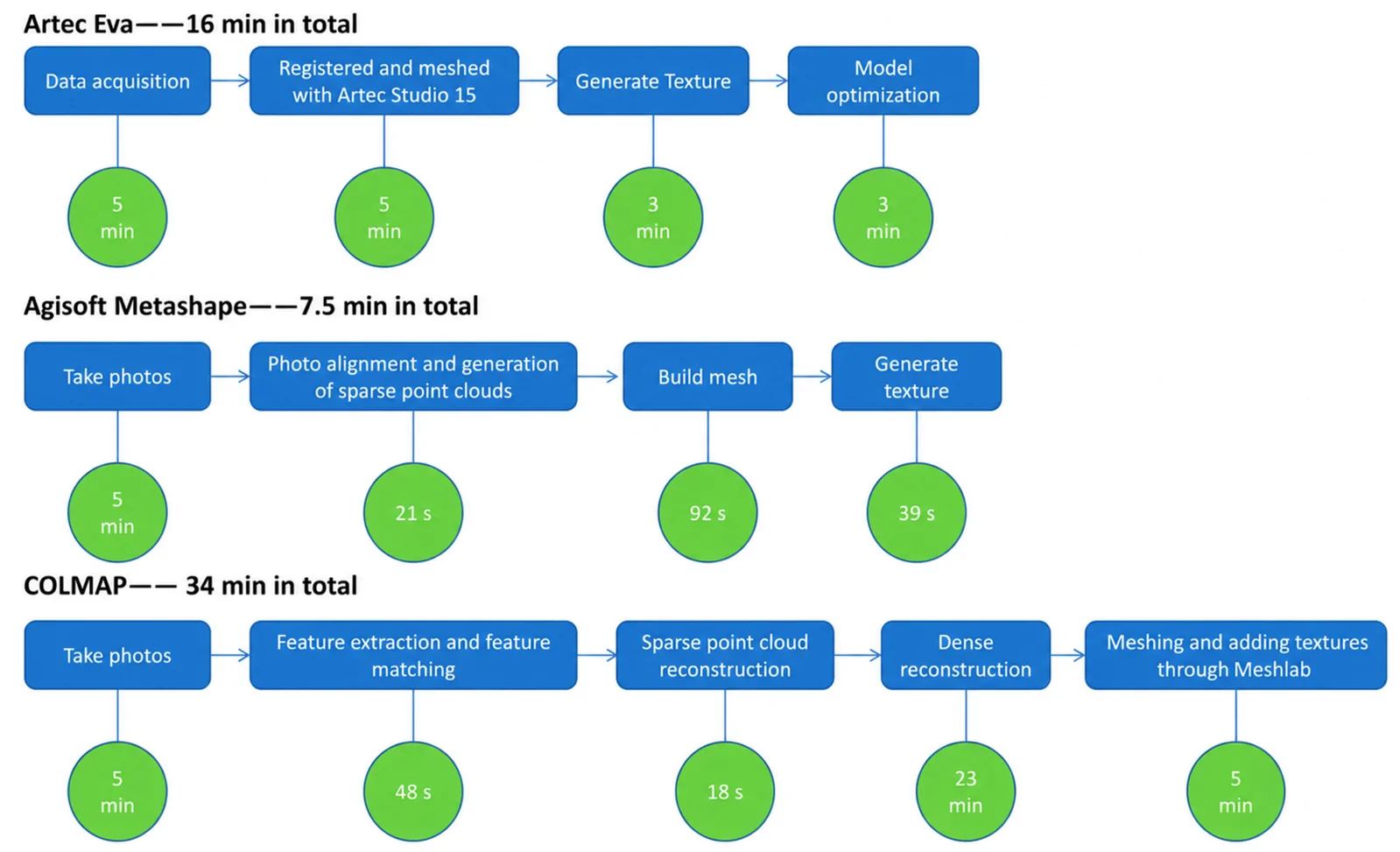
Workflow steps and approximate time requirements for three-dimensional model acquisition and reconstruction using Artec Eva, Agisoft Metashape, and COLMAP.

For the representative dataset, the approximate total workflow time was 16 min for Artec Eva, 7.5 min for Agisoft Metashape, and 34 min for COLMAP (Figure 3). These values are descriptive and were not subjected to statistical comparison because only one dataset was formally timed.

### 3.3. Intraclass Correlation Coefficients

Interrater reliability of RMS, MSD, and HD measurements was evaluated using intraclass correlation coefficients (ICCs). ICCs were calculated using a two-way mixed-effects model with absolute agreement. Because averaged measurements from the two investigators were used for subsequent analyses, average-measure ICCs were reported as the primary reliability estimates. All three parameters in both systems demonstrated excellent interrater reliability, with ICC values greater than 0.90. ICC estimates with 95% confidence intervals are presented in Table 1.

### 3.4. Normality of Paired Differences

The normality of paired differences between COLMAP and Agisoft Metashape was assessed using the Shapiro–Wilk test. The paired differences for HD (W = 0.815, *p* = 0.003) and MSD (W = 0.885, *p* = 0.038) significantly deviated from normality, whereas RMS showed a borderline result (W = 0.894, *p* = 0.053). Therefore, Wilcoxon signed-rank tests were used for inter-method comparisons of RMS, MSD, and HD.

### 3.5. Root Mean Square (RMS)

**Figure 4:**
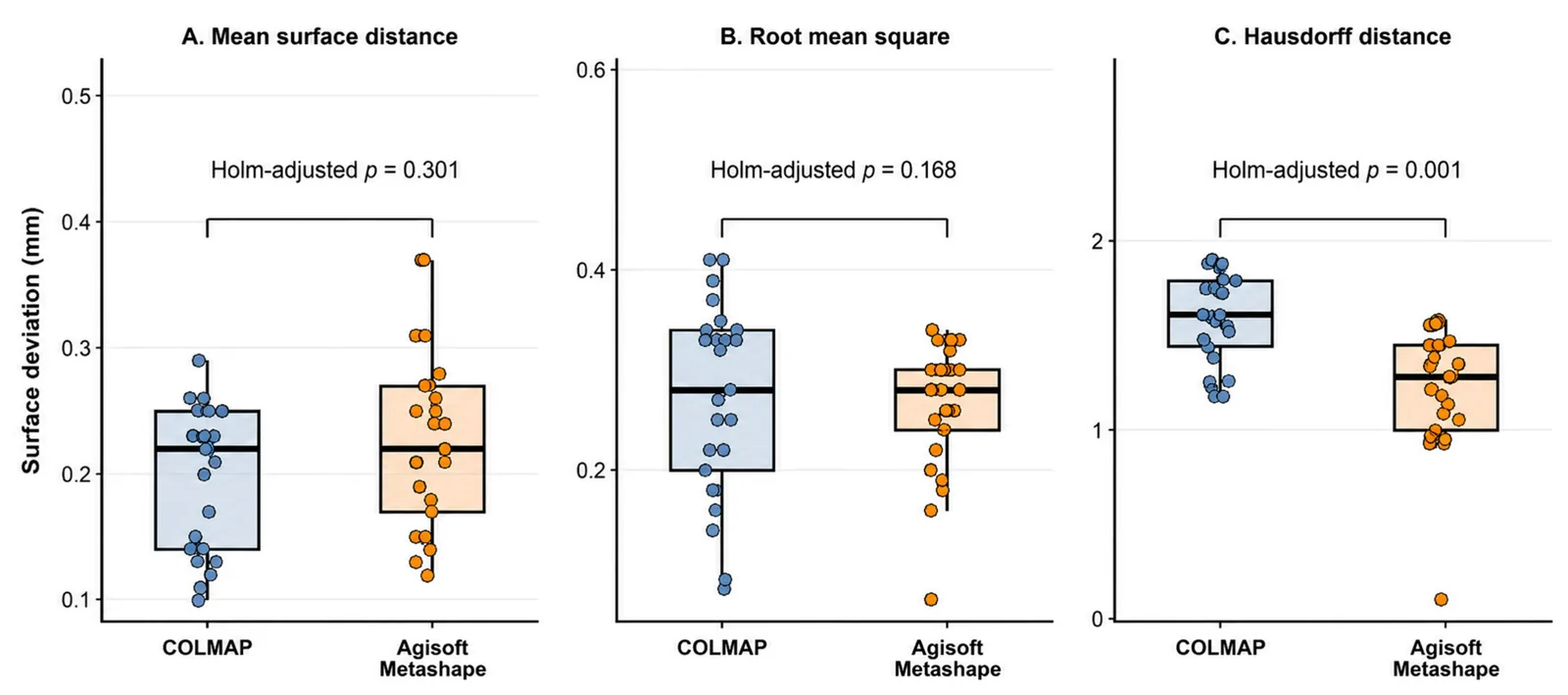
Model-to-model surface-deviation metrics for COLMAP- and Agisoft Metashape-derived reconstructions relative to the corresponding Artec Eva-derived models. (**A**) Mean surface distance (MSD, [mm]); (**B**) root mean square (RMS, [mm]); (**C**) Hausdorff distance (HD, [mm]).

The median RMS value was 0.28 mm (Figure 4; Table 2) for both Agisoft Metashape and COLMAP, and no statistically significant between-method difference was observed (*Z* = −1.728, unadjusted *p* = 0.084, Holm-adjusted *p* = 0.168, *n* = 25).

### 3.6. Mean Surface Distance (MSD)

The median MSD value was 0.22 mm (Figure 4; Table 2) for both methods, and no statistically significant between-method difference was observed (*Z* = −1.034, unadjusted *p* = 0.301; Holm-adjusted *p* = 0.301, *n* = 25).

### 3.7. Hausdorff Distance (HD)

Agisoft Metashape-derived reconstructions showed significantly lower HD values than COLMAP-derived reconstructions (Figure 4; Table 2; *Z* = −3.574, unadjusted *p* < 0.001 and Holm-adjusted *p* = 0.001, *n* = 25).

### 3.8. Bland–Altman Analysis

Bland–Altman analyses showed mean between-method differences of 0.030 mm for MSD, −0.008 mm for RMS, and −0.383 mm for HD. The corresponding 95% limits of agreement are presented in Supplementary Figure S2. Because the paired differences for MSD and HD were non-normally distributed, these findings were interpreted descriptively.

### 3.9. Surface-Distance Maps

**Figure 5:**
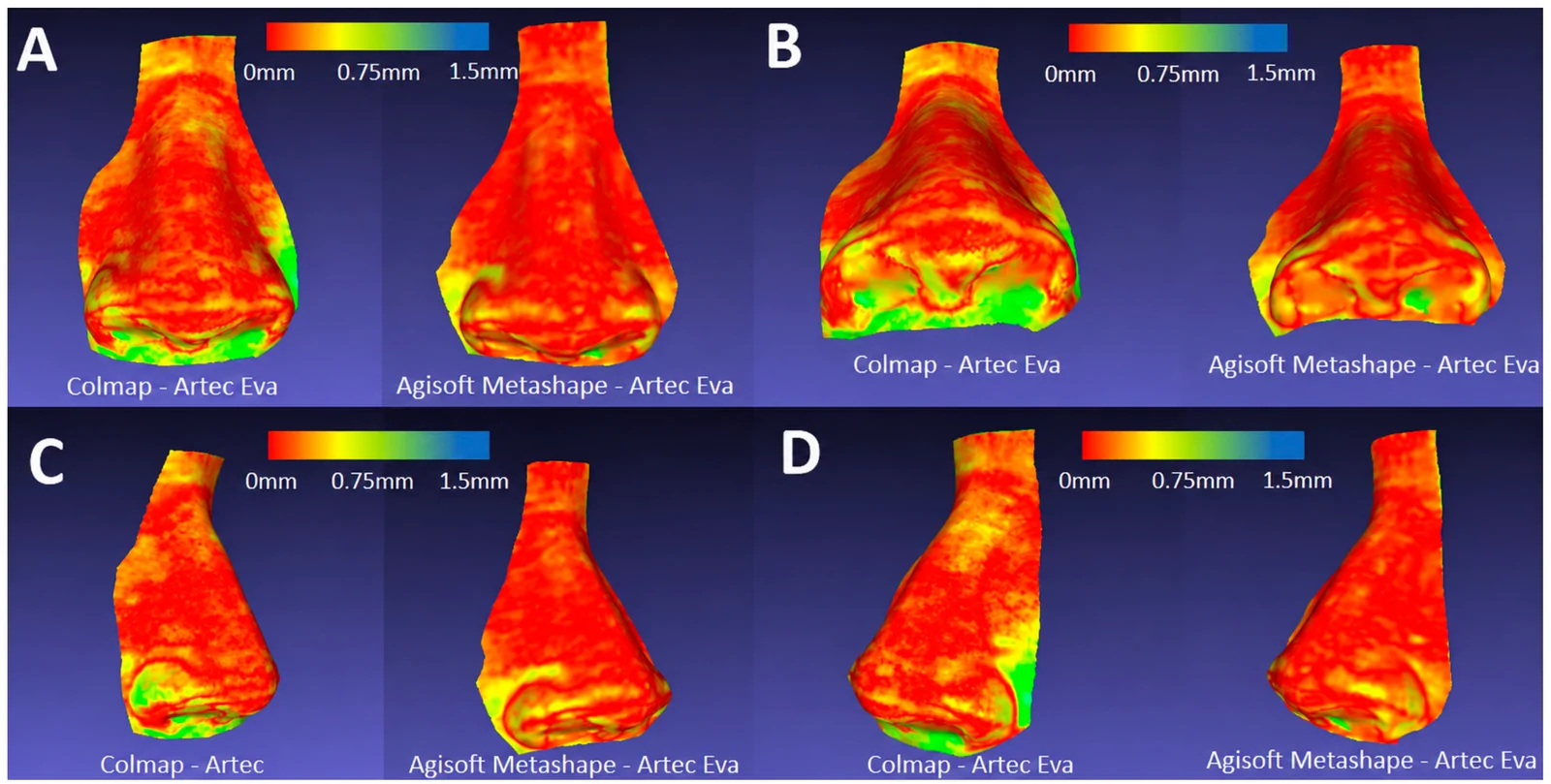
Surface-distance maps comparing COLMAP and Agisoft Metashape reconstructions with the Artec Eva model. (**A**) Front view; (**B**) bottom view; (**C**) right side view; (**D**) left side view.

Surface-distance heat maps showed generally small relative surface deviations between the passive stereophotogrammetry-derived models and the corresponding Artec Eva-derived comparator models. Figure 5 illustrates results from one representative participant: the left panel compares COLMAP with Artec Eva, and the right panel compares Agisoft Metashape with Artec Eva. The lowest surface deviations were mainly observed at the nasal tip and dorsum. In contrast, larger deviations were observed at the alae, the superior nasal root margin, and the alar-facial groove.

## 4. Discussion

In this study, we evaluated the relative surface-deviation performance and operational feasibility of two passive stereophotogrammetry-based 3D reconstruction methods using a previously evaluated structured-light handheld scanner as the reference comparator. Facial 3D models from 25 healthy participants were reconstructed with all three methods. Relative surface deviations were quantified in MeshLab using Hausdorff distance (HD), mean surface distance (MSD), and root mean square (RMS) values.

For HD, all models generated by COLMAP and Agisoft Metashape had values within 0.9–2.0 mm. Agisoft Metashape produced a significantly lower HD value overall (median 1.28 mm), reflecting smaller maximum local deviations than COLMAP, with an average inter-method difference of approximately 0.4 mm. As surface complexity increased, both methods showed larger maximum local discrepancies relative to Artec Eva. These trends are consistent with prior studies on COLMAP and Agisoft Metashape, which reported similar deviation magnitudes under comparable imaging conditions [28,29,30]. Notably, some models reconstructed by passive methods exhibited large local surface discrepancies. These local discrepancies may reflect noise artifacts in the passive reconstructions, uncertainty in the Artec Eva acquisition and reconstruction workflow, registration-related effects, minor participant motion between acquisitions, or a combination of these factors.

For RMS and MSD, both passive stereophotogrammetry-based methods showed small relative deviations from the Artec Eva-derived comparator models. Median RMS and MSD values for both methods were below 0.3 mm, and maximum values did not exceed 0.45 mm. To contextualize these findings, we compared the observed values with previously reported deviation ranges for 3D facial and nasal imaging. Ritschl et al. reported deviations from the reference model ranging from −1.23 to 1.57 mm in a comparative study of nasal 3D imaging systems [16]. Additional methodological and review studies have suggested that facial scanning deviations below 1.0 mm are commonly reported in three-dimensional facial imaging studies [31,32]. In the present study, the global deviation metrics of the passive workflows were within these previously reported ranges. However, these results should be interpreted as evidence of technical feasibility under standardized acquisition conditions rather than as direct evidence of clinical effectiveness in rhinoplasty patients. HD reflects maximum local deviation rather than global agreement and reached approximately 1–2 mm in some cases. Therefore, local deviations should be interpreted cautiously, particularly in anatomically complex regions such as the alae and adjacent perinasal soft tissues, where greater discrepancies were observed on surface-distance maps.

Preliminary testing suggested that passive stereophotogrammetry-based 3D reconstruction may be more sensitive to variations in acquisition conditions than active vision-based systems. Imaging conditions, subject motion, and camera angles all influenced reconstruction quality. In preliminary testing, images were captured with a handheld moving camera rather than with the finalized protocol, in which the camera remained fixed and the subject rotated on a motorized turntable. The handheld approach produced reconstructions with more noise artifacts and lower overall quality, largely due to inconsistent object distance and irregular angular intervals. Such inconsistencies complicate feature-point matching during registration and may reduce reconstruction quality.

Adequate photographic coverage is critical for reliable 3D reconstruction. Sufficient overlap and multi-angle views improve reconstruction stability, while exposure parameters strongly affect reconstruction outcomes. Key factors identified in this study were aperture and exposure control. In preliminary testing, apertures of f/8–f/11 provided adequate sharpness and depth of field for facial detail recovery. In contrast, larger apertures (e.g., f/1.8–f/2.0) reduced depth of field and created blurred regions. Manual exposure control is recommended over automatic settings, which may introduce inconsistent illumination across images and degrade texture quality. Uniform lighting is also essential. Two fill lights positioned at appropriate angles ensured even illumination, minimized shadows, and facilitated exposure optimization.

Although both passive methods require no specialized 3D measurement equipment and are therefore highly accessible, they differ in workflow integration and operational complexity. COLMAP performs feature extraction, pose registration, and point cloud generation, but its Poisson surface reconstruction function is limited. Selective removal of dense point clouds is not supported, so MeshLab is needed for manual cleaning, meshing, and texture mapping. When noise overlaps heavily with the target cloud, CloudCompare may be required for additional filtering. In contrast, Agisoft Metashape provides an integrated pipeline that automates processing from feature extraction to mesh and texture generation. Its built-in filters may suppress noise artifacts and improve reconstruction stability, and in this study, it showed lower maximum local deviations than COLMAP. However, these advantages come at a cost: the Professional edition is priced at USD 3499 and the Standard edition at USD 179. By comparison, COLMAP and its companion tools are open-source and free, offering a distinct cost advantage.

### Limitations

There are several limitations to this study that should be acknowledged. First, the sample size was limited, and no formal a priori sample size calculation was performed. Therefore, this study should be regarded as an exploratory methodological assessment, and the effect-size estimates should be confirmed in larger studies. Second, the cohort consisted only of healthy young Han Chinese adults aged 19–23 years, and no patients undergoing rhinoplasty, nasal reconstruction, or treatment for nasal deformity were included. Therefore, the findings should be interpreted as technical feasibility data from healthy participants rather than as evidence of clinical performance in surgical cases. Third, the Artec Eva scan was used as a reference comparator rather than an absolute ground truth. Therefore, the reported RMS, MSD, and HD values should be interpreted as relative surface deviations from a structured-light scanning workflow, not as absolute anatomical measurement errors. Future studies should include larger and more diverse cohorts, clinical nasal deformity cases, and higher-precision reference standards.

## 5. Conclusions

Under standardized acquisition conditions, COLMAP and Agisoft Metashape showed close relative agreement with Artec Eva-derived models for nasal three-dimensional surface reconstruction in healthy participants. Both passive stereophotogrammetry workflows achieved low global surface-deviation values, suggesting that image-based reconstruction may provide a feasible and accessible approach for standardized nasal surface measurement. Agisoft Metashape showed a lower value for Hausdorff distance than COLMAP, indicating smaller maximum local deviations and better control of localized surface errors. In contrast, COLMAP remains a free and open-source option, which may be valuable for users requiring a low-cost reconstruction workflow.

These findings support the technical feasibility of passive stereophotogrammetry for nasal surface reconstruction and provide a methodological basis for further evaluation in clinical settings. Future studies involving clinical cases, larger and more diverse cohorts, repeated acquisition sessions, and higher-precision reference standards are needed to determine its applicability in rhinoplasty or nasal reconstruction.

## Figures and Tables

**Table 1:** Interrater reliability of measurements obtained by two independent raters, assessed using intraclass correlation coefficients based on a two-way mixed-effects model.

Parameter	ICC	95% CI
COLMAP (HD)	0.989	0.972–0.996
Agisoft Metashape (HD)	0.978	0.942–0.992
COLMAP (MSD)	0.952	0.835–0.984
Agisoft Metashape (MSD)	0.933	0.851–0.962
COLMAP (RMS)	0.981	0.947–0.993
Agisoft Metashape (RMS)	0.982	0.952–0.993

**Table 2:** Summary statistics of surface-deviation metrics for COLMAP and Agisoft Metashape reconstructions relative to the Artec Eva models.

		COLMAP (HD)	Agisoft Metashape (HD)	COLMAP (MSD)	Agisoft Metashape (MSD)	COLMAP (RMS)	Agisoft Metashape (RMS)
Median (mm)		1.61	1.28	0.22	0.22	0.28	0.28
Minimum (mm)		1.18	0.93	0.10	0.12	0.08	0.07
Maximum (mm)		1.90	1.58	0.29	0.37	0.41	0.33
Percentiles (mm)	25	1.41	1.03	0.14	0.16	0.19	0.23
	50	1.61	1.28	0.22	0.22	0.28	0.28
	75	1.79	1.45	0.25	0.27	0.34	0.30
SD (mm)		0.235	0.224	0.057	0.070	0.096	0.054
Wilcoxon Z		−3.574		−1.034		−1.728	
Unadjusted *p*		<0.001		0.301		0.084	
Holm-adjusted *p*		0.001		0.301		0.168	

## Data Availability

Due to privacy and ethical restrictions, the facial 3D models generated and analyzed in this study are not publicly available, as they contain potentially identifiable participant information. The data-processing and analysis workflow supporting the findings of this study is provided in the Supplementary Materials. The underlying measurement data are not publicly available due to participant privacy and ethical restrictions but may be made available from the corresponding author upon reasonable request, subject to institutional and ethical approval.

## Supplementary Materials

The following supporting information can be downloaded at https://www.mdpi.com/article/10.3390/bioengineering13080899/s1. Figure S1: complete 39-image acquisition sequence and photographic coverage; Figure S2: Bland–Altman plots for RMS, MSD, and HD; File S1: Detailed reconstruction and registration steps.

## Abbreviations

The following abbreviations are used in this manuscript:

2D	Two-dimensional
3D	Three-dimensional
3DSI	Three-dimensional surface imaging
SfM	Structure from motion
MVS	Multi-view stereo
ICP	Iterative closest point
RMS	Root mean square
MSD	Mean surface distance
HD	Hausdorff distance
ICC	Intraclass correlation coefficient
CI	Confidence interval
SD	Standard deviation
OBJ	Object file format
