# Supplementary material for: Relative Agreement and Feasibility of Low-Cost Passive Stereophotogrammetry for Nasal Three-Dimensional Surface Imaging: A Comparison of COLMAP and Agisoft Metashape with a Handheld Structured-Light Scanner
Source: Bioengineering (Basel). 2026 Aug 9;13(8):899. doi: 10.3390/bioengineering13080899 (PMC13510088; doi:10.3390/bioengineering13080899)

Detailed reconstruction and registration Steps

COLMAP reconstruction steps are as follows：

1. Establish a new project.

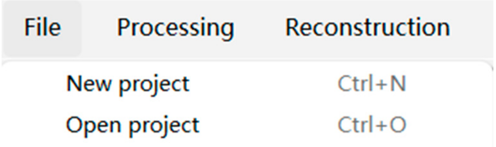

2. Feature extraction.

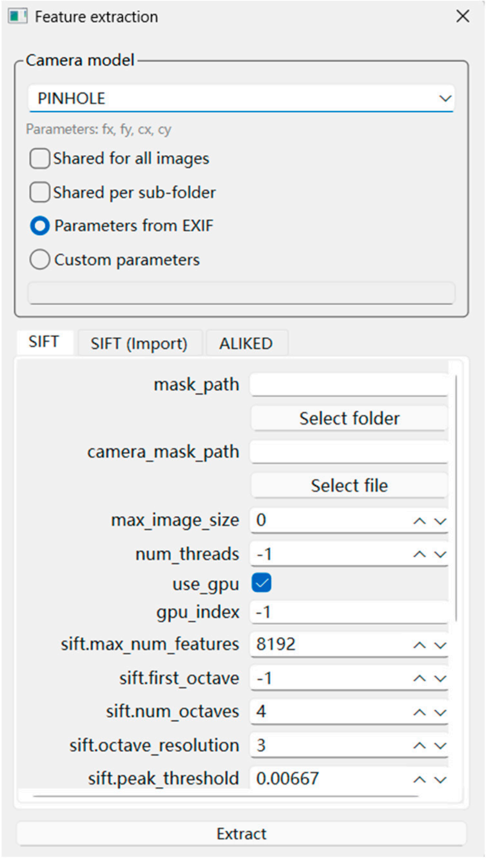

3. Feature matching.

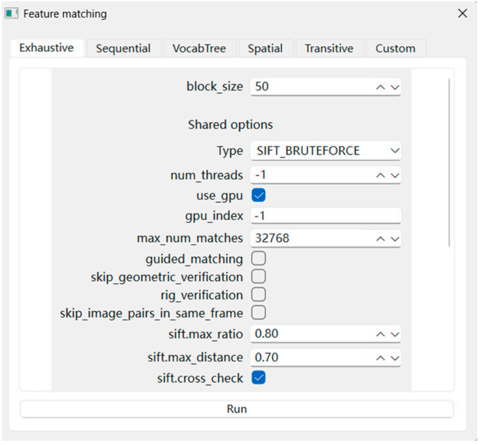

4. Reconstruction > start reconstruction.

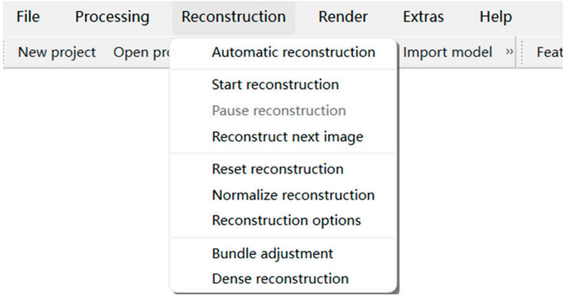

5. After sparse reconstruction was completed, the dense reconstruction workflow was initiated by selecting reconstruction > dense reconstruction. Three sequential operations were then performed: undistortion, stereo, and fusion.

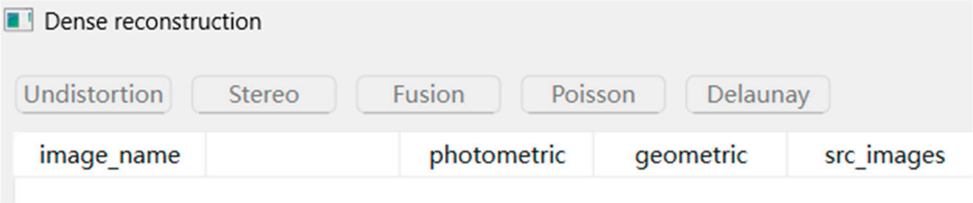

6. Export the dense point cloud as a .ply file and import it into MeshLab.

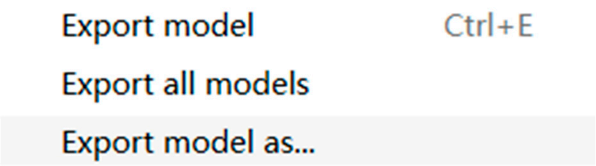

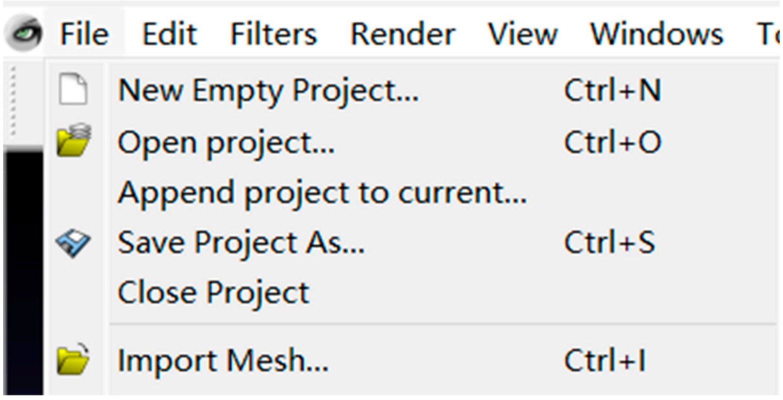

7. Use the selection and deletion tools to remove irrelevant noise points.

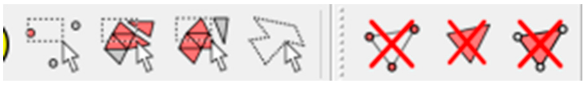

8. Meshing: Filter –> remeshing simplification and reconstruction –> screened Possion surface reconstruction.

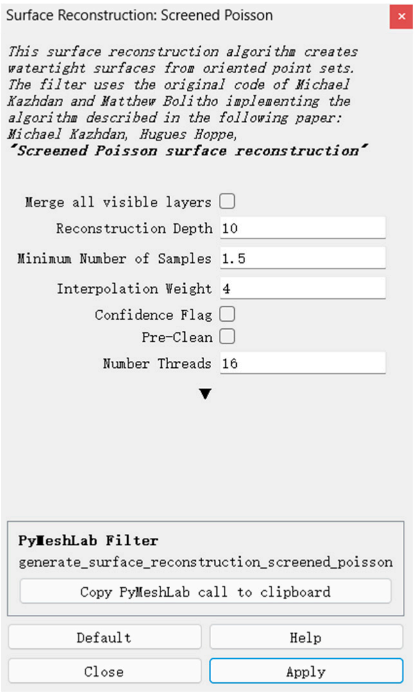

9. Delete the shell and excess parts generated by Poisson reconstruction: Filters –> cleaning and repairing–> remove isolated pieces.

10. Repair manifold edges: Filters–> selection –> select non-manifold edges.

11. Parameterization: Filter–> texture –> parameterization from registered rasters.

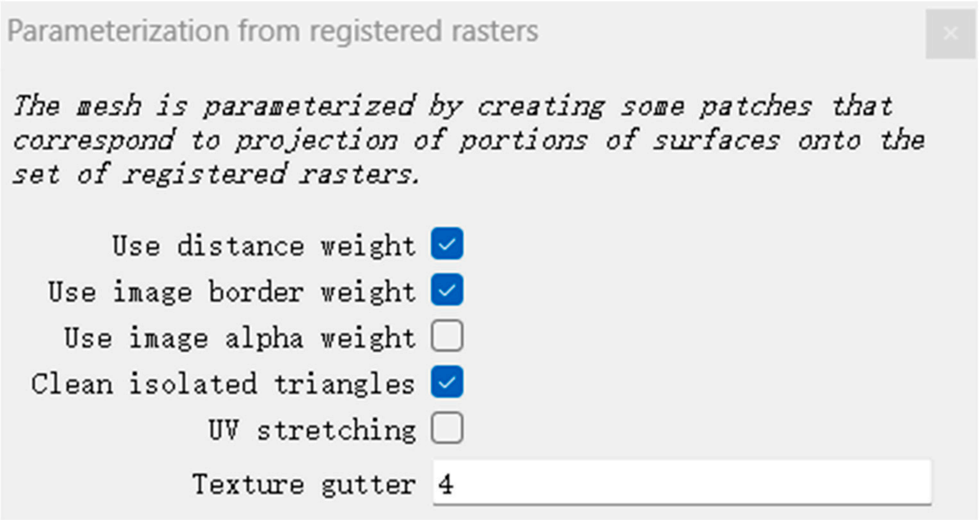

12. Project texture: Filter–> texture –> project-active rasters are colored to the current mesh, filling the texture.

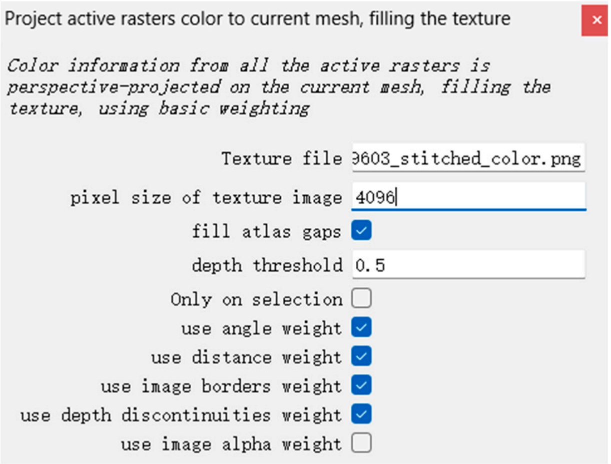

13. Export.

Agisoft metashape reconstruction steps are as follows：

1. Workflow—>add photos.
2. Workflow—>align photos.

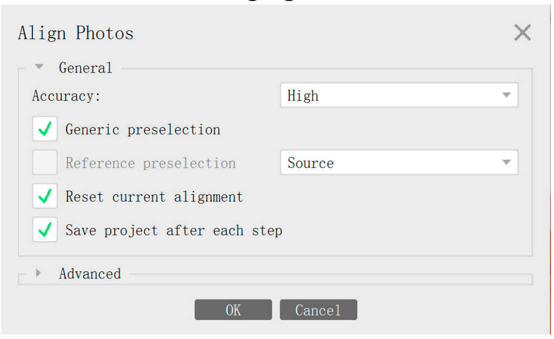

3. Workflow—>build model.

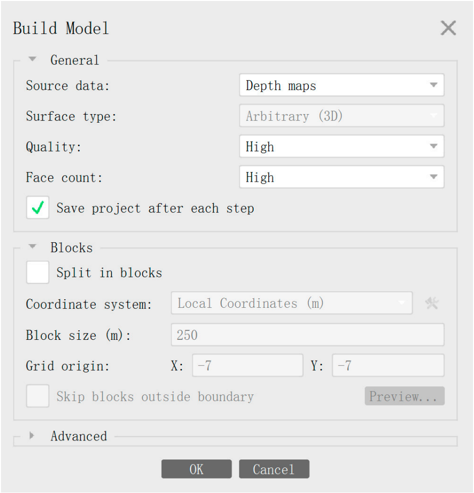

4. Workflow—>build texture.

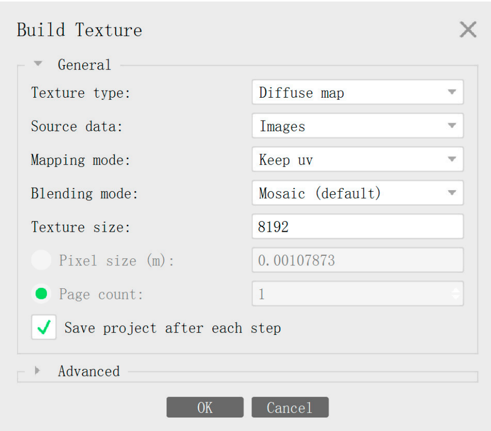

5. Export.

Artec Studio processing steps are as follows:

1. Quickly remove the background and any irrelevant areas: editor —> eraser.
2. Tools —> global registration —> apply.
3. Tools —> outlier removal —> apply.
4. Tools → sharp fusion and set the resolution to 0.5 mm.
5. Texture panel → export texture atlas → apply.
6. Export.

MeshLab registration and surface-distance analysis

1. Import the nasal model to be compared and the reference nasal model separately.

2. Enter the Align tool. Glue either one of the models, and then click the other model and select Point-Based Gluing.

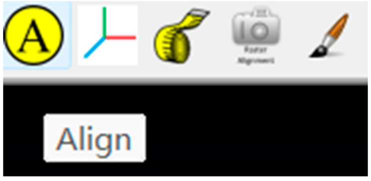

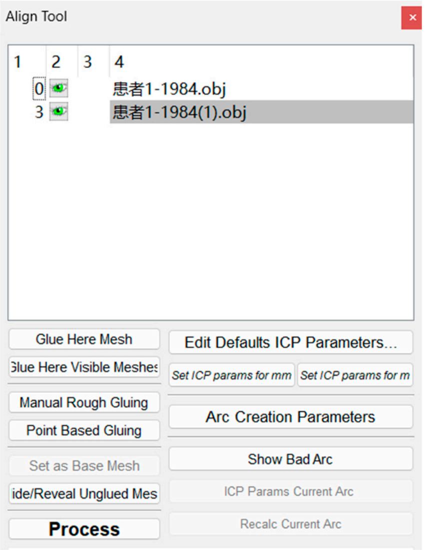

3. Use the left and right **Alar Base Junctions**, **Subnasale**, **nasal tip**, and **Nasion** as registration landmarks, and mark them one by one. After completing the initial registration, click **Process**.

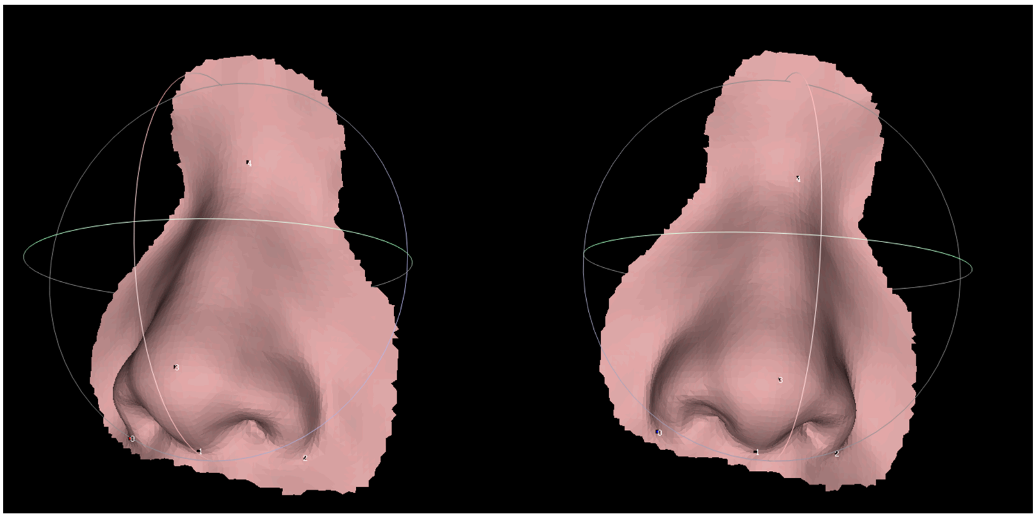

4. Filters—> sampling—>Hausdorff distance.

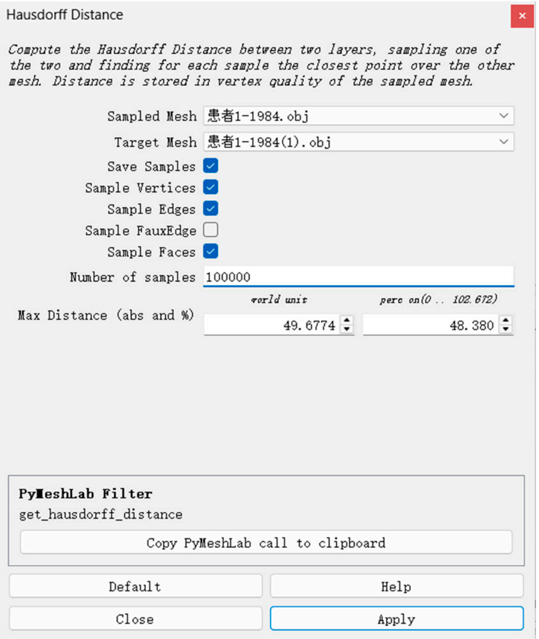

Supplement: Supplementary file 1 [file bioengineering-13-00899-s001.zip › bioengineering-4475890-supplementary-file S1.pdf]
